# Transitions from Ideal to Intermediate Cholesterol Levels may vary by Cholesterol Metric

**DOI:** 10.1038/s41598-018-20660-2

**Published:** 2018-02-09

**Authors:** Joseph C. Engeda, Katelyn M. Holliday, Shakia T. Hardy, Sujatro Chakladar, Dan-Yu Lin, Gregory A. Talavera, Barbara V. Howard, Martha L. Daviglus, Amber Pirzada, Pamela J. Schreiner, Donglin Zeng, Christy L. Avery

**Affiliations:** 1 Departments of Epidemiology, the University of North Carolina at Chapel Hill, Chapel Hill, NC USA; 2 Departments of Biostatistics, the University of North Carolina at Chapel Hill, Chapel Hill, NC USA; 3 Carolina Population Center, the University of North Carolina at Chapel Hill, Chapel Hill, NC USA; 4 Division of Health Promotion and Behavioral Science, San Diego State University, San Diego, CA USA; 5 MedStar Health Research Institute and Georgetown/Howard Universities Center for Clinical and Translational Sciences, Hyattsville, MD USA; 6 Institute for Minority Health Research, University of Illinois at Chicago, Chicago, IL USA; 7 Division of Epidemiology and Community Health, University of Minnesota, Minneapolis, MN USA

## Abstract

To examine the ability of total cholesterol (TC), a low-density lipoprotein cholesterol (LDL-C) proxy widely used in public health initiatives, to capture important population-level shifts away from ideal and intermediate LDL-C throughout adulthood. We estimated age (≥20 years)-, race/ethnic (Caucasian, African American, and Hispanic/Latino)-, and sex- specific net transition probabilities between ideal, intermediate, and poor TC and LDL-C using National Health and Nutrition Examination Survey (2007–2014; N = 13,584) and Hispanic Community Health Study/Study of Latinos (2008–2011; N = 15,612) data in 2016 and validated and calibrated novel Markov-type models designed for cross-sectional data. At age 20, >80% of participants had ideal TC, whereas the race/ethnic- and sex-specific prevalence of ideal LDL-C ranged from 39.2%-59.6%. Net transition estimates suggested that the largest one-year net shifts away from ideal and intermediate LDL-C occurred approximately two decades earlier than peak net population shifts away from ideal and intermediate TC. Public health and clinical initiatives focused on monitoring TC in middle-adulthood may miss important shifts away from ideal and intermediate LDL-C, potentially increasing the duration, perhaps by decades, that large segments of the population are exposed to suboptimal LDL-C.

## Introduction

The American Heart Association (AHA) “2020 Strategic Impact Goals for 2020 and Beyond” introduced the novel aim to improve cardiovascular health (CVH), which is measured by several indices, including total cholesterol (TC)^1^. High TC is a major risk factor for cardiovascular disease (CVD)^2^ and shows sex-and race/ethnic-specific patterning across the lifecourse^3^. Even though declines in mean TC have been observed over the past decades^4^, contemporary estimates suggest that 53.7% of United States (US) adults still do not meet the criteria for ideal TC^5^.

While TC is used frequently in health promotion initiatives, including AHA’s Life’s Simple 7^1^, Healthy People 2020^6^, and the SCORE Project^7^, it serves as a surrogate for low density lipoprotein (LDL-C)^8^. LDL-C, which carries most of the circulating cholesterol in the body and constitutes 60–70% of TC, is the primary target for cholesterol lowering treatment, with LDL-C lowering drug therapies shown to reduce CVD risk^9^. Although TC is easier to measure than LDL-C, as it can be assessed directly through a lipid panel^6^, few studies have evaluated how well TC transitions across adulthood mirror LDL-C transitions in contemporary diverse populations^10^, despite the utility of such research to inform the targeting of interventions to maintain or improve ideal cholesterol levels^11^. One possible reason why so few studies have contrasted TC and LDL-C transitions across adulthood among diverse populations is that such investigations would require decades-long longitudinal studies including multiple race/ethnicities to evaluate how cholesterol changes over adulthood. However, given strong secular trends in health behaviors and risk factors known to affect TC and LDL-C (e.g. physical activity, adiposity, and diet)^12–14^, results from longitudinal studies may not be generalizable to contemporary settings. Longitudinal studies also likely do not adequately capture populations who have undergone demographic shifts over the past decades, including Hispanic/Latinos. Therefore, using a novel extension to traditional Markov-type state transition models, we addressed these research gaps by estimating age, sex- and race/ethnic-specific *net* transition probabilities between ideal, intermediate, and poor cholesterol as measured by TC and LDL-C in African American, Caucasian, and Hispanic/Latino male and female populations using contemporary cross-sectional data^15^.

## Results

A maximum of 29,196 participants (14.6% African American; 32.0% Caucasian; 53.4% Hispanic/Latino) were included in this analysis (Table 1). At age 20, the prevalence of ideal TC ranged from 79.0% (Hispanic/Latino men) to 89.7% (Hispanic/Latino women), notably higher than the prevalence of ideal LDL-C, which ranged from 38.2% (Hispanic/Latino men) to 59.6% (African American women) (Supplementary Figs S1, S2). Additionally, to assess the influence of small changes in cholesterol levels on net transition probabilities, we evaluated TC and LDL-C medians (IQRs) and found the values were distinct from category-specific cutpoints (Supplementary Table S1).

**Table 1:** Race/ethnic- and sex-specific demographics for NHANES and HCHS/SOL participants.

Characteristic	African American		Caucasian		Hispanic/Latino	
	Women	Men	Women	Men	Women	Men
Total cholesterol (TC)						
N.	2191	2051	4721	4621	9405	6207
^*^Median age (IQR)	44 (32,56)	44 (30,56)	50 (36,63)	49 (35,61)	43 (31,54)	41 (30,52)
^*†^Median TC (IQR)	188 (162,214)	184 (158,213)	197 (172,226)	188 (162,216)	191 (166,221)	194 (167,223)
^†^TC prevalence at age 20						
% Ideal	84.5	81.9	85.4	83.4	89.7	79.0
% Intermediate	13.6	16.0	11.8	13.3	9.4	17.9
% Poor	1.9	2.1	2.8	3.3	0.9	3.1
^‡^% Lipid- lowering medication use	15.0	15.0	18.2	20.7	11.0	8.9
^§^Low density lipoprotein (LDL-C)						
N.	968	834	2144	2018	9,277	5,985
^*^Median age (IQR)	43 (32, 55)	42 (29,53)	49 (37,62)	49 (36,61)	42 (31,54)	41 (30,52)
^*†^Median LDL-C (IQR)	111 (99,129)	108 (89,134)	116 (95,139)	114 (93,134)	116 (94,142)	121 (145,97)
^†^LDL-C prevalence at age 20						
% Ideal	59.6	50.9	54.8	51.4	54.1	38.2
% Intermediate	25.9	23.3	32.9	30.3	36.7	41.6
% Poor	14.5	25.7	12.3	18.3	9.2	20.2
^‡^% Lipid- lowering medication use	14.6	13.5	16.7	20.8	11.0	8.9

^*^IQR, interquartile range.

^†^Smoothed prevalence proportions.

^‡^Population wide estimates.

^§^Included only blood samples ≥8 hours fasting.

### Net transition probabilities from ideal to intermediate to poor TC

Net transition probability patterns from ideal to intermediate TC across adulthood varied by race/ethnicity and sex. For example, net transition probabilities from ideal to intermediate TC peaked earlier for men than women, with peak net transition probabilities among men occurring between age 36 (African American and Caucasian) and 37 (Hispanic) (Supplementary Fig. S3). For example, a net 2.5% (95% CI: 1.6%, 3.5%) of African American men aged 36 years of age with ideal TC transitioned to intermediate TC by age 37. For women, peak ideal to intermediate TC net transition probabilities occurred between 42 (Caucasian) and 57 years (African American) of age.

In contrast to ideal to intermediate TC net transition probabilities that suggested distinct net transition probability patterns by sex that peaked in middle adulthood, net transition probabilities from intermediate to poor TC peaked approximately 15 years earlier and differed more by race/ethnicity than sex (Supplementary Fig. S4). For example, net transition probabilities from intermediate to poor TC peaked at age 20, the youngest age under investigation, for both Caucasian men (net transition probability = 3.6% [95% CI: 2.5%, 4.7%]) and Caucasian women (net transition probability = 3.0% [95% CI: 2.0%, 3.9%]). In contrast, intermediate to poor TC net transition probabilities were approximately equivalent through age 40 for African Americans and Hispanic/Latinos.

### Net transition probabilities from ideal to intermediate to poor LDL-C

In contrast to ideal to intermediate TC net transition probabilities that peaked between 36–57 years of age, net transition probabilities from ideal to intermediate LDL-C peaked considerably earlier; by age 29 for men (age range: 26–29) and age 46 for women (age range: 32–46) (Supplementary Fig. S5).

Liken to net transition results from intermediate to poor TC, all net transition probabilities from intermediate to poor LDL-C also peaked before age 40 (age range: 20–36), although intermediate to poor LDL-C net transition probabilities were approximately equivalent for African Americans and Caucasians (Supplementary Fig. S6). Also, intermediate to poor LDL-C net transition probabilities in African American men and women were notably less precise than intermediate to poor LDL-C net transition probabilities estimated in Caucasian and Hispanic/Latino men and women.

### Population Extrapolations

**Figure 1:**
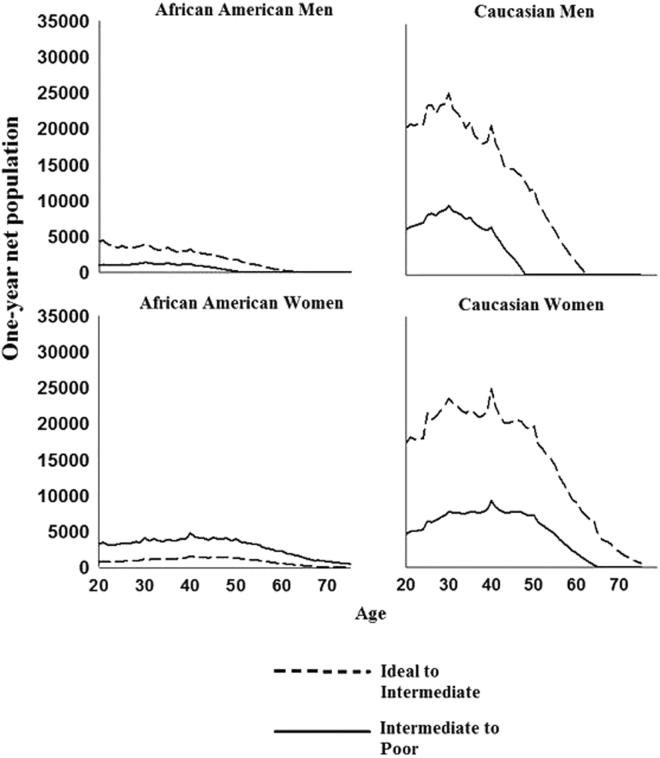
One-year population extrapolations of the net population transitions from ideal-intermediate and intermediate-poor TC*.

**Figure 2:**
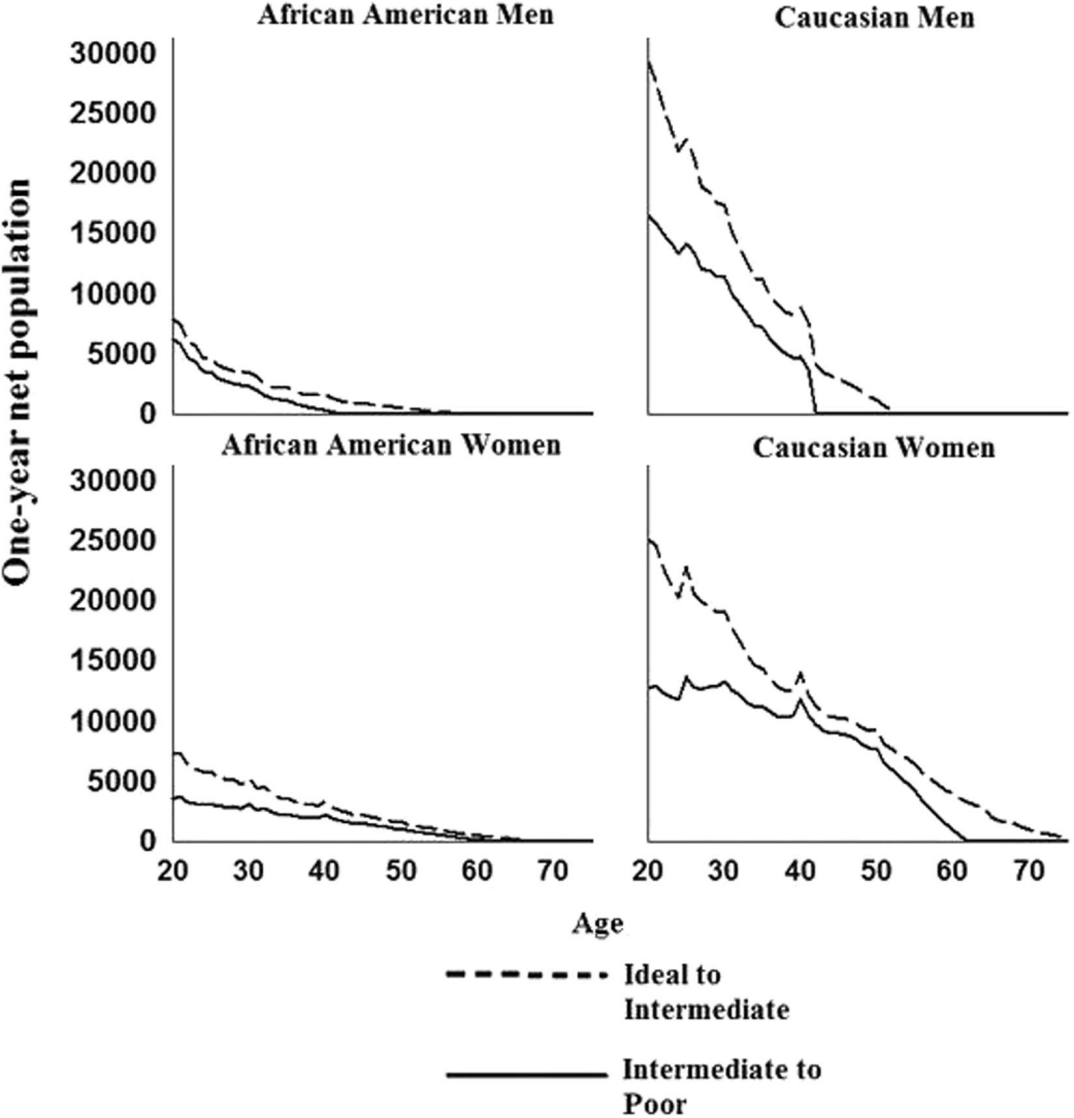
One-year population extrapolations of the net population transitions from ideal-intermediate and intermediate-poor LDL-C*.

Finally, we extrapolated results to Caucasian and African American 2010 civilian noninstitutionalized men and women aged 20 years and greater. As described above, extrapolating age-, sex-, and race/ethnic-specific net transition probabilities allowed us to combine estimates of relative effect size (in example net transition probabilities) with population size estimates and ideal and intermediate TC and LDL-C prevalence estimates, facilitating examination of the one-year age-specific net number of African American and Caucasian men and women transitioning to intermediate and poor TC and LDL-C. Overall, net population extrapolations demonstrated considerable variation between TC and LDL-C in the ages associated with the largest shifts away from ideal and intermediate TC and LDL-C. For example, in contrast to ideal to intermediate TC net population extrapolations, which suggested that the largest one-year net population shifts occurred in mid-life for African Americans and Caucasians, these same net population extrapolations for LDL-C peaked at age 20, after which steep declines were observed (Figs 1 and 2). Results for intermediate to poor TC and LDL-C showed a slightly different picture that varied by sex; here we focused on Caucasians given limited precision of estimated net transition probabilities in African Americans. Specifically, among Caucasian men, the largest net population shifts from intermediate to poor LDL-C occurred at age 20, again in contrast with one-year net population extrapolations estimated for intermediate to poor TC, which suggested that net population transitions from intermediate to poor TC were approximately constant through age 40. For Caucasian women, TC and LDL-C intermediate to poor net population extrapolations were consistent, suggesting approximately constant age-specific net population transitions until 40 years of age, when declines in LDL-C net transitions were observed. Equivalent declines in TC were not observed until 10 years later at 50 years of age. Additionally, extrapolation to the 2010 Hispanic/Latino population within HCHS/SOL study center census tracts displayed high variability between net population increases, likely due to the smaller sample size of census tract populations (Supplementary Fig. S7), although TC and LDL-C net population extrapolations remained constant through age 40 for both men and women.

## Discussion

In this study, we showed that net population shifts toward intermediate and poor LDL-C levels in adulthood peaked approximately 20 years earlier than net population shifts toward intermediate and poor TC. Variation by sex was also observed, with the magnitude of decline in intermediate and poor LDL-C net population shifts occurring more rapidly for men than women. Overall, these results suggest public health and preventive medicine initiatives monitoring TC in mid-adulthood may miss important shifts away from ideal and intermediate LDL-C that occur as early as age 20.

Although past studies have found TC and LDL-C to be highly correlated^16^, TC levels are easier to collect compared to LDL-C^17^. Yet, as we demonstrated herein, focusing on TC as a surrogate to LDL-C could direct population-wide interventions and goals toward midlife. Although consistent with prior longitudinal research examining TC transitions in mid-adulthood^18^, this focus would target populations approximately one to two decades after peak net population shifts away from ideal and intermediate LDL-C occurred. Reliance on TC to monitor and direct initiatives therefore has the potential to increase the duration, perhaps by decades, that large segments of the population are exposed to suboptimal LDL-C levels, increasing CVD risk^19^.

Re-evaluation of public health efforts to maintain ideal levels of LDL-C throughout adulthood are therefore warranted, especially among younger male populations for whom net transitions away from ideal and intermediate LDL-C are highest, but are less likely to receive routine medical care including routine cholesterol screening^20^. Indeed, the American Heart Association/American College of Cardiology (AHA/ACC) recommended that risk reduction should begin as early as possible in order to effectively reduce lifetime risk of CVD, suggesting every adult ≥20 years of age receive cholesterol screenings every five years. However, the low levels of preventive service utilization highlight the need to strengthen preventive efforts beyond the clinic and before LDL-C levels become elevated, especially in light of adverse effects of prolonged exposure to even moderate elevations in TC and LDL-C on CVD risk (in example primordial prevention)^19^. Dietary and lifestyle interventions are key components of such primordial prevention initiatives. For example, the AHA/ACC Guidelines on Lifestyle Management to Reduce Cardiovascular Risk^21^ endorsed dietary patterns such as the DASH dietary pattern, which lowered LDL-C by 11 mg/dl (9.3% relative change from baseline) compared to an American diet in the 1990s^21,22^. Indeed, for every 1% reduction in LDL-C levels, dietary trials have shown the relative risk for major CVD events is reduced by approximately 1%^22^, emphasizing that even modest LDL-C reductions represent a meaningful and effective goal if sustained over the course of early life^23,24^. Public health initiatives to reduce LDL-C are not limited to adult populations. For example, the Special Turku Coronary Risk Factor Intervention Project (STRIP) initiated low saturated fat diets and parental counseling for infants and observed lower LDL-C levels in the intervention group compared to the control group at multiple follow-up time points spanning 3 years to 19 years^23,24^, which is relevant given the modest prevalence of ideal LDL-C observed at the beginning of adulthood.

Additionally, increased primordial prevention is important for populations with intermediate LDL-C, given that large segments of the population transition into poor LDL-C, have difficulties retaining intermediate or ideal levels, and are ineligible for treatment under current guidelines^25^. Previous clinical trials have shown diet and exercise interventions to reduce LDL-C by up to 11 mg/dl in trial settings among older participants and those with poor LDL-C levels^22^. While promoting healthier lifestyles may delay the progression of elevated LDL-C levels and shift large segments of the population back to healthier LDL-C levels, the degree to which modest population-wide lifestyle shifts could delay net transitions away from ideal LDL-C and subsequently elevated CVD risk deserves further evaluation.

It is important to note that our results demonstrating the divergence between population-wide LDL-C and TC net population shifts complement rather than oppose current cholesterol treatment guidelines. For example, current cholesterol treatment guidelines recommended by the American College of Cardiology/American Heart Association suggest evaluating traditional clinical risk factors, while recommendations from the European Society of Cardiology/European Atherosclerosis Society also consider factors such as elevated triglyceride, social deprivation, central obesity, elevated lipoprotein (a), subclinical atherosclerosis, or family history of CVD as part of a global risk score to calculate total CVD risk^25,26^. In contrast to cholesterol treatment guidelines that assess CVD risk for treatment purposes, our study highlights populations most at-risk and the most appropriate metric to monitor LDL-C levels to prevent peak net population shifts away from ideal LDL-C. The biggest gains in primordial prevention will occur from targeting young adults before net population shifts away from ideal LDL-C occur to ensure populations maintain ideal levels and delay exposure to elevated CVD risk.

Past research examining cholesterol through the life course has been limited by short follow-up periods^4^, examination of specific life epochs (in example childhood)^10^, use of non-contemporary data^27^, or restrictions to largely Caucasian populations^28^. Our present study was developed to respond to these limitations and instead utilized contemporary, population-based studies of multiethnic populations that spanned early to late adulthood, enabled by the application of novel statistical methodology^15^. Additionally, we limited examination to one-year net transition probabilities in an attempt to avoid introducing biases from age-specific factors (in example reasons for immigration) that could influence longer-term age-specific effects. Furthermore, we conducted the analyses separately for each race/ethnicity and reported results for Hispanic/Latino men and women separately from African American and Caucasian men and women, reducing the extent differences between studies may have influenced results. Yet, several limitations must be noted. Our study did not examine children because age-specific changes in cholesterol profiles across childhood may reflect screening inaccuracies during pubertal years. Second, while it might be useful to quantitate the extent to which transitions are accounted for by small increments in TC and LDL-C, it would likely require longitudinal data to calculate the individual transitions instead of the net transition probabilities from cross-sectional data presented by this study. Third, although net transition probabilities highlight important population level movements, however, they may not be well-suited for examining the complex interplay between cholesterol sub-fractions (LDL-C, high density lipoprotein, and triglycerides), motivating future studies that evaluate these metrics longitudinally. Fourth, we were unable to evaluate the influence of other factors that may affect net transition probabilities (in example diet^9^ or physical activity^14^) as this examination would require further stratification of our data, likely beyond the ability of the current sample sizes.

Using TC as an indicator for LDL-C may miss population shifts toward intermediate and poor LDL-C that occur in early adulthood. Population-wide initiatives that maintain ideal levels of LDL-C in early adulthood through lifestyle changes can be an effective approach to prevent elevated levels of LDL-C and subsequent downstream CVD events and deserve further evaluation.

## Methods

### Study Populations

We characterized the ages at which African American, Caucasian, and Hispanic/Latino populations transitioned between ideal, intermediate, and poor TC and LDL-C using cross-sectional data from two population-based studies: the Hispanic Community Health Study/Study of Latinos (HCHS/SOL)^29^ and the National Health and Nutrition Examination Survey (NHANES)^30^. The following review boards approved the study: Institutional Review Board (IRB) of Albert Einstein College of Medicine (Montefiore Medical Center), IRB of Feinberg School of Medicine at Northwestern University, IRB of Miller School of Medicine at the University of Miami, and the San Diego State University IRB. In addition, all participants in both NHANES and HCHS/SOL provided informed consent and methods were performed in accordance with the relevant guidelines and regulations.

The HCHS/SOL is a population-based cohort designed to examine risk and protective factors for chronic diseases and to quantify morbidity and mortality prospectively^29^. At study baseline (2008 to 2011), 16,415 Hispanic/Latino men and women aged 18–76 years who self-identified as Hispanic/Latino were enrolled from sampled households in four US communities (Bronx, NY; Chicago, IL; Miami, FL; San Diego, CA). For this study, we evaluated n = 15,612 HCHS/SOL participants aged 20–75 years of age at baseline.

NHANES includes demographic, nutritional, and health status information on a nationally representative biennial probability sample of the US civilian population (aged 0–80+ years)^30^. For this study, we used data from the four most contemporary NHANES population cross-sections (2007–2014) and evaluated Caucasian and African American populations aged 20–75 years. We excluded non-Hispanic Asian participants who were available as of the 2011–12 population cross-section as well as participants who identified as “Mexican American”, “Other Hispanic” or “Other Race – Including Multi-Racial” due to small sample sizes, particularly for Hispanic/Latino populations when compared to the HCHS/SOL study.

### Measurement and classification of TC and LDL-C

TC (mg/dl) was measured using a cholesterol oxidase enzymatic method and characterized using AHA defined cutpoints as ideal (<200 mg/dl untreated), intermediate (200–239 mg/dl or treated to goal), or poor (≥240 mg/dl)^1^. Although AHA did not define ideal, intermediate, and poor LDL-C, we used the National Cholesterol Education Program (NCEP) Adult Treatment Panel III categories^17^. Briefly, LDL-C (mg/dl),measured among participants reporting fasting for at least eight hours, was calculated using the Friedewald equation, and was characterized as ideal (<100 mg/dl untreated), intermediate (100–130 mg/dl or treated to <100 mg/dl), or poor (>130 mg/dl)^31^. Medication data were collected using medication inventories. Participants were asked to provide containers for medications taken in the last 30 days. If any of the medications reported were lipid-lowering medications, the participants were classified as current lipid-lowering medication users.

### Statistical analysis

#### Estimation of net transition probabilities

We characterized the ages at which African American, Caucasian, and Hispanic/Latino populations transitioned between ideal, intermediate, and poor TC and LDL-C categories using cross-sectional data from HCHS/SOL and NHANES, and statistical models that estimate one-year net transition probabilities^15^. For example, in a longitudinal study of 100 participants with ideal TC aged 20 years, if 10 participants transitioned from ideal to intermediate TC by age 21 and three participants transitioned from intermediate to ideal TC by age 21, the ideal-to-intermediate TC net transition would be seven participants; dividing by the number of ideal TC participants at age 20 (n = 100) would yield the net transition probability, which in this case is 7%. The intermediate to ideal TC net transition is defined as 0 because fewer participants moved from intermediate to ideal TC than from ideal to intermediate TC^15^. Under the assumption that the age-specific TC and LDL-C transitions probabilities remained approximately stable across time (see Supplementary Information), we can use cross-sectional data to estimate net transition probabilities; estimation of the number of participants transitioning from ideal to intermediate TC and intermediate to ideal TC (in example individual transition probabilities) would require longitudinal data.

Estimation of net transition probabilities from cross-sectional data required three steps. First, we estimated the age-, sex-, and race/ethnic-specific prevalence of each category (in example ideal; intermediate; poor) using a multinomial logit model^32,33^ and P-spline smoothing^34^. Cluster sampling methods were used to accommodate family structure (HCHS/SOL) and complex sampling designs (HCHS/SOL and NHANES). Next, we used a series of simplex algorithms from linear programming theory to estimate age-, sex-, and race/ethnic-specific net transition probabilities (see Supplementary Information). Results were then examined and showed exceptional calibration and validation (see Supplementary Information). Finally, bootstrapping was used to estimate 95% confidence intervals (95% CI), where the age-specific prevalence was simulated from its asymptotic distribution and net transition probabilities were computed for each simulated prevalence^15^.

Finally, we extrapolated our results to African American and Caucasian 2010 civilian, non-institutionalized U.S. population and to the 2010 Hispanic/Latino population within HCHS/SOL community census tracts to estimate the net population of individuals transitioning between TC and LDL-C levels. Extrapolation was accomplished by multiplying the age, sex-, and race/ethnicity-specific net transition probabilities (e.g., ideal to intermediate) by the prevalence of the initial TC and LDL-C level (e.g., ideal) and the appropriate age-, race/ethnicity-, and sex-specific 2010 population sizes. Statistical analyses were performed using SAS (Cary, NC), STATA (College Station, TX), and R (Vienna, Austria). All data analyses took place in 2016.

### Data availability

The NHANES dataset generated during and analyzed during the current study is available from the corresponding author on reasonable request; however, HCHS/SOL dataset cannot be made publically available without consent from the HCHS/SOL study.

## Electronic supplementary material

Supplementary Information
