# Author Correction: Transitions from Ideal to Intermediate Cholesterol Levels may vary by Cholesterol Metric

**DOI:** 10.1038/s41598-018-24108-5

**Published:** 2018-04-13

**Authors:** Joseph C. Engeda, Katelyn M. Holliday, Shakia T. Hardy, Sujatro Chakladar, Dan-Yu Lin, Gregory A. Talavera, Barbara V. Howard, Martha L. Daviglus, Amber Pirzada, Pamela J. Schreiner, Donglin Zeng, Christy L. Avery

**Affiliations:** 1 Departments of Epidemiology, the University of North Carolina at Chapel Hill, Chapel Hill, NC USA; 2 Departments of Biostatistics, the University of North Carolina at Chapel Hill, Chapel Hill, NC USA; 3 Carolina Population Center, the University of North Carolina at Chapel Hill, Chapel Hill, NC USA; 4 Division of Health Promotion and Behavioral Science, San Diego State University, San Diego, CA USA; 5 MedStar Health Research Institute and Georgetown/Howard Universities Center for Clinical and Translational Sciences, Hyattsville, MD USA; 6 Institute for Minority Health Research, University of Illinois at Chicago, Chicago, IL USA; 7 Division of Epidemiology and Community Health, University of Minnesota, Minneapolis, MN USA

Correction to: *Scientific Reports* 10.1038/s41598-018-20660-2, published online 09 February 2018

The Acknowledgements section in this Article is incomplete.

“The Coronary Artery Risk Development in Young Adults Study (CARDIA) is conducted and supported by the National Heart, Lung, and Blood Institute (NHLBI) in collaboration with the University of Alabama at Birmingham (HHSN268201300025C & HHSN268201300026C), Northwestern University (HHSN268201300027C), University of Minnesota (HHSN268201300028C), Kaiser Foundation Research Institute (HHSN268201300029C), and Johns Hopkins University School of Medicine (HHSN268200900041C). CARDIA is also partially supported by the Intramural Research Program of the National Institute on Aging (NIA) and an intra-agency agreement between NIA and NHLBI (AG0005). This manuscript has been reviewed by CARDIA for scientific content. This work was supported by National Heart, Lung, and Blood Institute training grant T32 predoctoral fellow T32HL007055.”

should read:

“The authors thank the staff and participants of HCHS/SOL (Investigators website - http://www.cscc.unc.edu.libproxy.lib.unc.edu/hchs/), NHANES, and the Coronary Artery Risk Development in Young Adults Study (CARDIA) for their important contributions. CARDIA is conducted and supported by the National Heart, Lung, and Blood Institute (NHLBI) in collaboration with the University of Alabama at Birmingham (HHSN268201300025C & HHSN268201300026C), Northwestern University (HHSN268201300027C), University of Minnesota (HHSN268201300028C), Kaiser Foundation Research Institute (HHSN268201300029C), and Johns Hopkins University School of Medicine (HHSN268200900041C). CARDIA is also partially supported by the Intramural Research Program of the National Institute on Aging (NIA) and an intra-agency agreement between NIA and NHLBI (AG0005). This manuscript has been reviewed by CARDIA for scientific content. This work was supported by National Heart, Lung, and Blood Institute training grant T32 predoctoral fellow T32HL007055.”

