# Supplementary material for: Transitions from Ideal to Intermediate Cholesterol Levels may vary by Cholesterol Metric
Source: Sci Rep. 2018 Feb 9;8:2782. doi: 10.1038/s41598-018-20660-2 (PMC5807429; doi:10.1038/s41598-018-20660-2)
Supplement: Supplementary file 1 — Supplementary Information [file 41598_2018_20660_MOESM1_ESM.pdf]

SUPPLEMENTARY INFORMATION

Transitions from Ideal to Intermediate Cholesterol Levels may vary by Cholesterol Metric

Joseph C Engeda, MPH*[1]; Katelyn M Holliday[2], PhD; Shakia T Hardy[3], PhD; Sujatro Chakladar[4], MPH; Dan-Yu Lin[5], PhD; Gregory A Talavera[6], PhD; Barbara V Howard[7], PhD; Martha L Daviglus[8], MD, PhD; Amber Pirzada[9], MD; Pamela J Schreiner[10], PhD; Donglin Zeng[11], PhD; Christy L Avery[12], PhD

From the Departments of Epidemiology[1,2,3,12], Biostatistics[4,5,11], and Carolina Population Center[12], the University of North Carolina at Chapel Hill, NC; Division of Health Promotion and Behavioral Science, San Diego State University[6], CA; Center for the Study of Sex Differences in Health, Aging & Disease, Georgetown University Medical Center[7], DC; Department of Medicine Institute for Minority Health Research, University of Illinois at Chicago[8,9], IL; Division of Epidemiology and Community Health, University of Minnesota[10], MN.

The supporting information has the following sections in order:

I. Estimation of net transition probabilities 3

II. Calibration of net transition probabilities 4

III. Validation of net transition probabilities 8

IV. Supplemental Tables 10

V. Supplemental Figures 13

VI. Supplementary references 20

I. Estimation of net transition probabilities

Our approach to estimating net transition probabilities was built upon a foundation established by operations research, allowing us to view the estimation of age-specific net transition probabilities between cholesterol levels as a transportation problem. Briefly, the transportation problem was conceptualized by specifying supplies, demands, shipping costs, decision variables, and an objective function. The supplies were interpreted as smoothed prevalence proportions $\hat{\pi}_i(a-1)$, where *a* represented age and *i* indexed the cholesterol level at age *a-1*, the demands were the prevalence proportions one year later $\hat{\pi}_j(a)$, *j* indexed the cholesterol level at age *a*, the cost constants were specified beforehand (c_{ij}, see below), the decision variables were the net transitions ($\tau'_{ij}(a)$, to be calculated) and the objective function *J* kept track of the total transportation costs between cholesterol levels at age *a-1* to cholesterol levels at age *a*. The objective function *J* was then minimized: $min\ J = \sum_{i=1}^{3}\sum_{j=1}^{3} c_{ij}\tau'_{ij}(a)$, subject to the conditions $\sum_{j=1}^{3}\tau'_{ij}(a) = \hat{\pi}_i(a-1), \sum_{i=1}^{3}\tau'_{ij}(a) = \hat{\pi}_j(a)$ and $\tau'_{ij}(a) \geq 0$. The conditions ensured that the total flow from cholesterol level *i* represented the supply of that level, that the total flow into cholesterol level *j* was equal to the demand of that level, the overall supply always equalled the overall demand, and that no transitions were negative. The net transition probabilities $p'_{ij}(a)$ were then estimated as $p'_{ij}(a) = \tau'_{ij}(a)/\hat{\pi}_i(a-1)$.

II. Calibration of net transition probabilities

Overview. Estimation of net transition probabilities requires the specification of a cost constant, c_{ij}, yet no study has attempted to calibrated cost constraints that describe movement within and between cholesterol levels using longitudinal data.

Approach. Initially we assigned zero costs to remaining within the same cholesterol level, a cost of one unit for moving one level up or down (e.g. ideal TC to intermediate TC), and a cost of three units for moving two levels up or two levels down (e.g. ideal TC to poor TC), as previously recommended.[1] This initial approach was then contrasted with cost constraints calculated using longitudinal data. Specifically, to calibrate cost constraints, we used longitudinal data from the Coronary Artery Risk Development in Young Adults (CARDIA) study, a multi-center, population-based study of the evolution of coronary heart disease risk beginning in young adulthood.[2] CARDIA investigators recruited n=5,115 African American (51.5%) and Caucasians (48.5%) males and females aged 18-30 at study baseline (1985-1986) from four US communities. Although CARDIA data are not ideal to describe contemporary cholesterol transitions given the large secular changes in cholesterol levels that have occurred over the past three decades and the restriction to African American and Caucasians populations, they can inform on the likelihood of moving between cholesterol levels.

To calibrate the cost constraints, we first estimated the age-specific cumulative probabilities for ideal (0), intermediate (1), and poor TC (2) as θ_0, θ_1 and θ_2 =1, respectively, using a cumulative logistic mixed effects model.

$$\theta_0(age,b) = \frac{\exp(\alpha_0 + \beta_1 \times age + \beta_2 \times age^2 + \gamma_1 \times I(race = white) + \gamma_2 \times I(sex = female) + \gamma_3 \times I(race = white, sex = female) + b)}{1 + \exp(\alpha_0 + \beta_1 \times age + \beta_2 \times age^2 + \gamma_1 \times I(race = white) + \gamma_2 \times I(sex = female) + \gamma_3 \times I(race = white, sex = female) + b)}$$

$$\theta_1(age,b) = \frac{\exp(\alpha_1 + \beta_1 \times age + \beta_2 \times age^2 + \gamma_1 \times I(race = white) + \gamma_2 \times I(sex = female) + \gamma_3 \times I(race = white, sex = female) + b)}{1 + \exp(\alpha_1 + \beta_1 \times age + \beta_2 \times age^2 + \gamma_1 \times I(race = white) + \gamma_2 \times I(sex = female) + \gamma_3 \times I(race = white, sex = female) + b)}$$

Here, α_0. α_1, β_1, β_2, γ_1, γ_2 and γ_3 are fixed effects and b are random effects assumed to follow a normal distribution with mean zero. The probabilities of ideal, intermediate, and poor TC given b are respectively:

$$\pi_0 = \theta_0, \pi_1 = \theta_1 - \theta_0, \pi_2 = 1 - \theta_1.$$

The prevalence for cholesterol category j at age $(a-1)$ is

$$\int_{-\infty}^{\infty} \pi_j\left(a-1,b\right) \times \frac{1}{\sqrt{2\pi\sigma}} \exp\left(-\frac{b^2}{2\sigma^2}\right) db$$

and the net transition from group j to group i for $j \neq i$ is

$$max(\int_{-\infty}^{\infty} \pi_i(a,b) \times \pi_j\left(a-1,b\right) \times \frac{1}{\sqrt{2\pi\sigma}} \exp\left(-\frac{b^2}{2\sigma^2}\right) db$$

$$-\int_{-\infty}^{\infty} \pi_j(a,b) \times \pi_i\left(a-1,b\right) \times \frac{1}{\sqrt{2\pi\sigma}} \exp\left(-\frac{b^2}{2\sigma^2}\right) db, 0)$$

For $i = j$, the net transition from group i to i at age a is defined as

prevalence of group i at age (a-1) - $\sum_{k \neq i}$ net transition from group i to k.

The net transition probability at age a from cholesterol category i to cholesterol category j is then defined as:

$$\frac{\text{net transition from i to j at age a}}{\text{Prevalence of group i at age a}}$$

The net transition probability is then obtained for each of the four race-sex groups and then averaged over the four groups.

We then used a numerical integration approach to calculate the integrals described in the above formulas.[3] Now we have net transition probabilities from the cross sectional model using the optimization algorithm approach for a given set of cost parameters and also net transition probabilities from the longitudinal model obtained by fitting the cumulative logistic mixed effects model. Calibration errors were calculated as $\sum_{age} \sum_{BMI\ groups} |$ net transition probability obtained from the cross sectional model - net transition probability obtained from the longitudinal model|.

The net transition probability obtained from the cross sectional model were based on the baseline value only. The cost parameters were searched in [0,20] with an increment of one, ensuring that the cost of remaining in the same group were less than that of transitioning to a different group and the cost of transitioning from category 0 to category 2 or category 2 to category 0 were larger than the sum of the other two cost parameters. The optimal cost parameters were then chosen as the parameters for which the calibration error was minimum.

Results. Our results showed that net transitions estimated using optimized cost constraints of 2, 8, and 15 calculated using longitudinal CARDIA data produced net transitions and standard errors that differed on average less than 0.01% from net transitions estimated using initial cost

constraints (0, 1, and 3), suggesting little influence of cost constraint definition on the estimation of net transitions or associated standard errors.

III. Validation of net transition probabilities

Overview. To evaluate the assumption that cholesterol levels transitions remained approximately stable across time, we compared observed cholesterol level prevalence proportions calculated from the 2011-2012 NHANES data with estimated 2011-2012 cholesterol level prevalence proportions calculated from net transition probabilities generated from 2007-2008 NHANES data. Overlap between observed and estimated cholesterol level prevalence proportions in 2007-2008 and 2011-2012 would suggest that cholesterol level transitions remained approximately stable across the time period of examination, here approximately four years.

Approach. First, we calculated age-specific cholesterol level net transition probabilities using the 2007-2008 NHANES data in the race/ethnic- and sex-combined African American and Caucasians sample using the approach described in the main paper.[1] The 2007-2008 net transition probabilities were then used to estimate the prevalence of ideal, intermediate, and poor TC four years (corresponding to the 2011-2012 NHANES data) later. Specifically, to estimate the expected age-specific prevalence of ideal, intermediate, and poor TC in 2011-2012 from 2007-2008 data, we defined an age *a* population of n_{a0} ideal TC participants, n_{a1} intermediate TC participants, and n_{a2} poor TC participants in 2007-2008. At age *a*, the four year net transition probability from cholesterol level i to cholesterol level j was p_{aij}. The four year net transition probability was obtained by multiplying the one year net transition probability matrix with itself for each age. We then:

I. Estimated $x_j = (x_{j0}, x_{j1}, x_{j2})$, the expected number of participants in moving from cholesterol level j to each cholesterol level (including j) by 2011-2012 as $n_{aj} \times (p_{aj0}, p_{aj1}, p_{aj2})$ for j in $\{0,1,2\}$.

II. The proportion of participants in each cholesterol level in 2011-2012 (indexed by *k*) is given as $= \frac{y_{ak}}{\sum_{i=0}^{2} y_{ai}}$ where $y_{ak} = \sum_{j=0}^{2} x_{jk}$.

III. Finally, we used locally weighted scatterplot smoothing (LOESS) to smooth the prevalence proportions across age.

Results. The results of the simulation are summarized in the following plot comparing expected and observed prevalence proportions in the population. From the plot we can see that the curve of the smoothed expected and observed proportion behave very similarly and are very close to each other which suggests that the cross sectional method performs well.

IV. Supplemental Tables

Supplementary Table 1. Selected age-, race/ethnic-, and sex-specific TC net transition probabilities

		Estimated net transition probabilities (95% confidence interval)					
		African Americans		Caucasians		Hispanic/Latino	
Transition	Age	Females	Males	Females	Males	Females	Males
Ideal-intermediate	20	1.1 (0.9,.3)	1.6 (1.3,1.8)	1.5 (1.3,1.7)	1.8 (1.7,2.0)	1.1 (0.9,1.2)	1.9 (1.7,2.2)
	30	1.8 (1.4,2.3)	2.4 (1.7,3.1)	2.8 (2.5,3.0)	3.2 (2.9,3.4)	2.3 (1.4,3,3)	3.9 (2.5,3.4)
	40	2.5 (1.9,3.2)	2.5 (1.4,3.5)	3.6 (3.2,4.0)	3.3 (2.9,3.7)	3.8 (0.7,7.0)	2.9 (2.5,3.3)
	50	3.0 (2.1,3.8)	1.6 (0.7,2.5)	3.5 (3.1,3.8)	2.1 (1.7,2.4)	4.5 (0.0,9.3)	1.4 (1.0,1.9)
	60	3.0 (1.9,4.2)	0.4 (0.0,1.2)	2.5 (2.1,2.9)	0.4 (0.0,0.8)	3.8 (0.0,9.0)	0.0 (0.0,0.3)
	70	2.8 (1.1,4.5)	0.0 (0.0,0.3)	1.1 (0.3,1.9)	0.0 (0.0,0.0)	2.6 (0.0,7.6)	0.0 (0.0,0.0)
Intermediate-poor	20	1.5 (0.6,2.3)	1.8 (0.6,3.1)	3.0 (2.0,3.9)	3.6 (2.5,4.7)	1.7 (0.0,3.4)	2.7 (1.7,3.7)
	30	1.6 (0.5,2.8)	2.0 (0.0,4.1)	2.6 (2.0,3.2)	2.9 (2.5,3.4)	2.8 (0.0,7.5)	3.3 (2.7,4.0)
	40	1.5 (0.0,3.0)	1.2 (0.0,3.6)	1.8 (1.2,2.3)	1.2 (0.9,1.6)	3.0 (0.0,12.0)	2.1 (1.6,2.6)
	50	1.0 (0.0,2.7)	0.1 (0.0,1.7)	0.9 (0.5,1.3)	0.0 (0.0,0.0)	2.0 (0.0,11.5)	0.0 (0.0,0.2)
	60	0.4 (0.0,2.0)	0.0 (0.0,0.6)	0.3 (0.0,0.5)	0.0 (0.0,0.0)	0.5 (0.0,8.3)	0.0 (0.0,0.0)
	70	0.0 (0.0,1.2)	0.0 (0.0,0.1)	0.0 (0.0,0.1)	0.0 (0.0,0.0)	0.0 (0.0,6.2)	0.0 (0.0,0.0)

NHANES, National Health and Nutrition Examination Survey.

Supplementary Table 2. Selected age-, race/ethnic-, and sex-specific LDL-C net transition probabilities

		Estimated net transition probabilities (95% confidence interval)					
		African Americans		Caucasians		Hispanic/Latino	
Transition	Age	Females	Males	Females	Males	Females	Males
Ideal-intermediate	20	2.8 (2.1,3.5)	3.8 (2.1,5.4)	3.0 (2.4,3.6)	4.5 (4.0,4.9)	2.8 (2.2,3.4)	3.4 (2.3,4.6)
	30	3.9 (2.7,5.0)	4.4 (0.0,9.0)	4.1 (3.1,5.1)	5.4 (4.6,6.2)	4.2 (3.5,4.9)	4.3 (3.2,5.4)
	40	4.0 (2.5,5.5)	3.5 (0.0,10.4)	4.3 (3.4, 5.2)	4.2 (3.4,5.0)	5.1 (4.6,5.6)	3.5 (2.8,4.2)
	50	3.1 (0.7,5.5)	1.7 (0.0,9.9)	3.5 (2.9,4.1)	1.8 (1.2,2.4)	5.1 (4.3,5.9)	1.6 (0.8,2.3)
	60	1.7 (0.0,5.1)	0.0 (0.0,9.4)	2.2 (1.2,3.1)	0.0 (0.0,0.1)	4.3 (2.7,5.9)	0.0 (0.0,0.8)
	70	0.2 (0.0,4.1)	0.0(0.0,10.1)	0.6 (0.0,2.0)	0.0 (0.0,0.0)	3.3 (0.7,5.9)	0.0 (0.0,1.1)
Intermediate-poor	20	2.0 (1.0,3.1)	4.3 (0.0,14.0)	1.7 (1.1,2.4)	3.0 (2.2,3.8)	1.7 (1.2,2.1)	2.6 (2.0,3.1)
	30	2.2 (0.2, 4.2)	2.6 (0.0,27.2)	2.0 (1.6,2.5)	1.9 (1.4,2.5)	2.5 (2.3,2.8)	3.4 (2.9,3.9)
	40	1.6 (0.0,5.3)	0.7 (0.0,40.2)	1.7 (1.3,2.2)	0.7 (0.3,1.1)	2.6 (2.2,3.0)	2.0 (1.5,2.5)
	50	0.6 (0.0,5.6)	0.0 (0.0,50.2)	1.0 (0.6,1.3)	0.0 (0.0,0.0)	1.7 (1.2,2.2)	0.0 (0.0,0.0)
	60	0.0 (0.0,5.5)	0.0 (0.0,59.1)	0.1 (0.0,0.,3)	0.0 (0.0,0.0)	0.3 (0.0,0.9)	0.0(0.0,0.0)
	70	0.0 (0.0,5.7)	0.0 (0.0,64.3)	0.0 (0.0,0.0)	0.0 (0.0,0.0)	0.0 (0.0,0.1)	0.0 (0.0,0.0)

NHANES, National Health and Nutrition Examination Survey.

Supplementary Table 3. Demographics for NHANES and HCHS/SOL participants used to characterize the age-specific net transition probabilities

	African American		Caucasian		Hispanic/Latino	
Characteristic	Women	Men	Women	Men	Women	Men
Total cholesterol (TC)						
Median TC (IQR)						
Ideal	166 (148-182)	164 (145-181)	171 (155-187)	167 (149-184)	172(155-186)	170(152-186)
Intermediate	209 (195-222)	209 (184-221)	210 (195-223)	205 (173-219)	212(202-224)	211(201-224)
Poor	259 (246-277)	257 (248-277)	260 (247-275)	258 (247-274)	260(249-281)	261(248-279)
*Median TC (IQR)						
Ideal	166 (148-182)	164 (145-181)	171 (155-187)	167 (149-184)	172(155-186)	170(152-186)
Intermediate	214 (207-225)	215 (207-224)	217 (208-227)	215 (207-226)	216(207-226)	216(207-226)
Poor	260 (247-277)	258 (249-278)	260 (248-275)	259 (248-273)	259(248-279)	260(248-278)
Low density lipoprotein (LDL-C)						
Median LDL-C (IQR)						
Ideal	84 (78-92)	84 (78-91)	87 (80-94)	87 (79-94)	88(81-94)	88 (81-94)
Intermediate	112 (105-121)	112 (103-121)	112 (103-121)	111 (103-120)	113(105-121)	114(105-122)
Poor	149 (140-165)	152 (140-166)	152 (141-169)	148 (138-165)	153 (141-172)	152(140-170)
*Median LDL-C (IQR)						
Ideal	84 (78-92)	84 (78-91)	87 (80-94)	87 (79-94)	88(81-94)	88 (81-94)
Intermediate	115 (107-122)	115 (106-123)	115 (108-123)	115 (108-122)	115(107-122)	116(108-123)
Poor	148 (140-165)	152 (140-165)	151 (141-168)	148 (138-166)	152(140-171)	152(140-170)

* Non-medicated populations
IQR, interquartile range

V. Supplemental Figures

Supplementary Figure 1. Smoothed age (20-75)-, race/ethnic-, and sex-specific prevalence proportions of ideal, intermediate, and poor TC

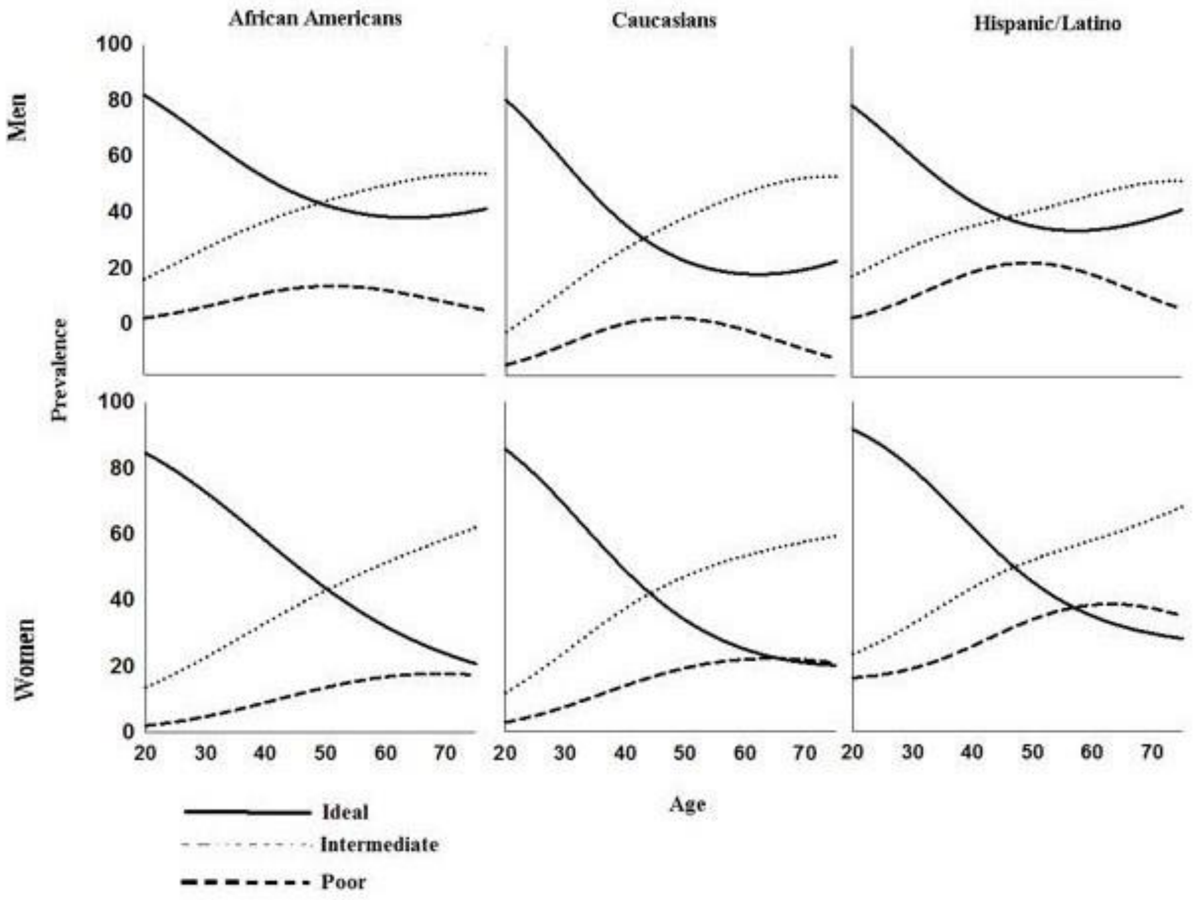

Supplementary Figure 2. Smoothed age (20-75)-, race/ethnic-, and sex-specific prevalence proportions of ideal, intermediate, and poor LDL

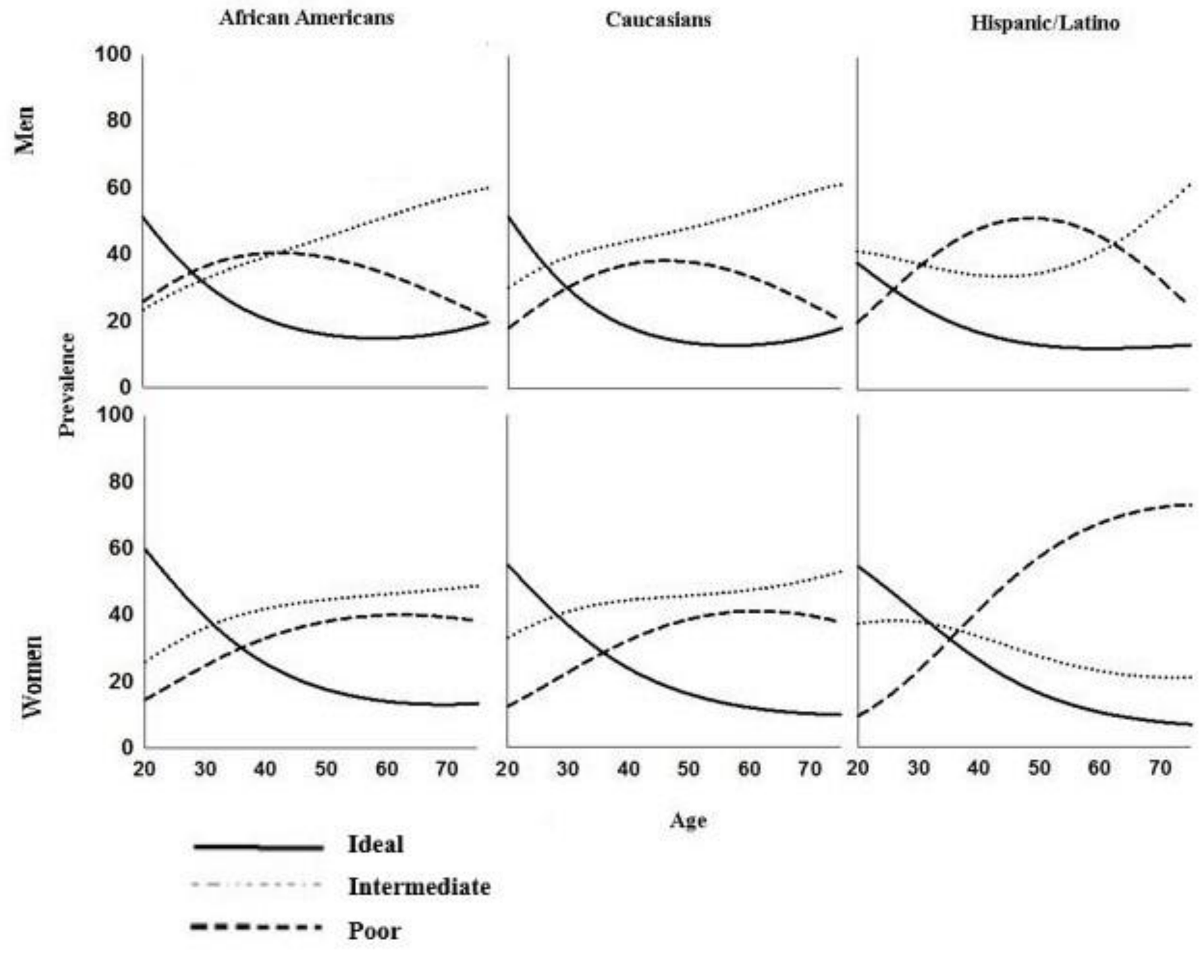

Supplementary Figure 3. Age (20-75)-, race/ethnic-, and sex-specific ideal TC to intermediate TC net transition probabilities

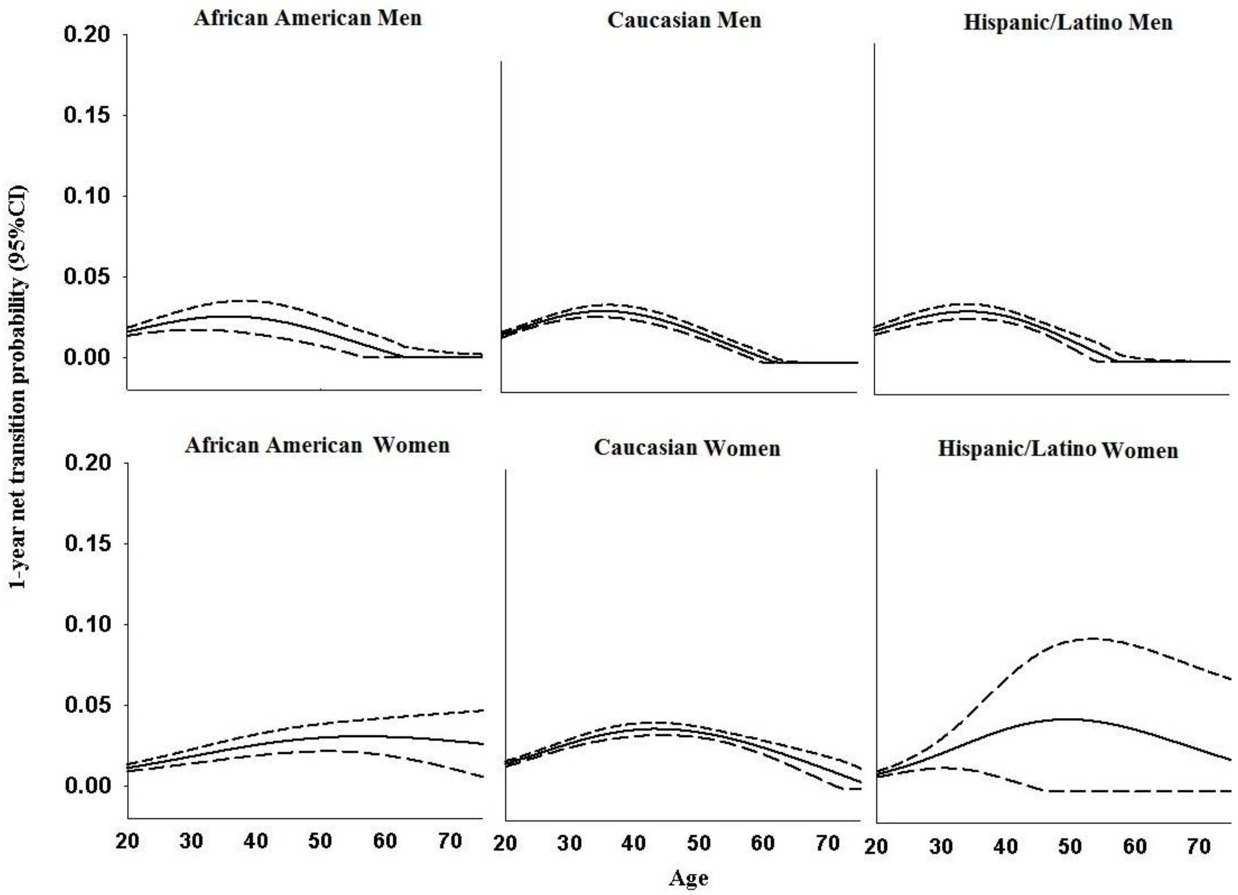

Supplementary Figure 4. Age (20-75)-, race/ethnic-, and sex-specific intermediate TC to poor TC net transition probabilities

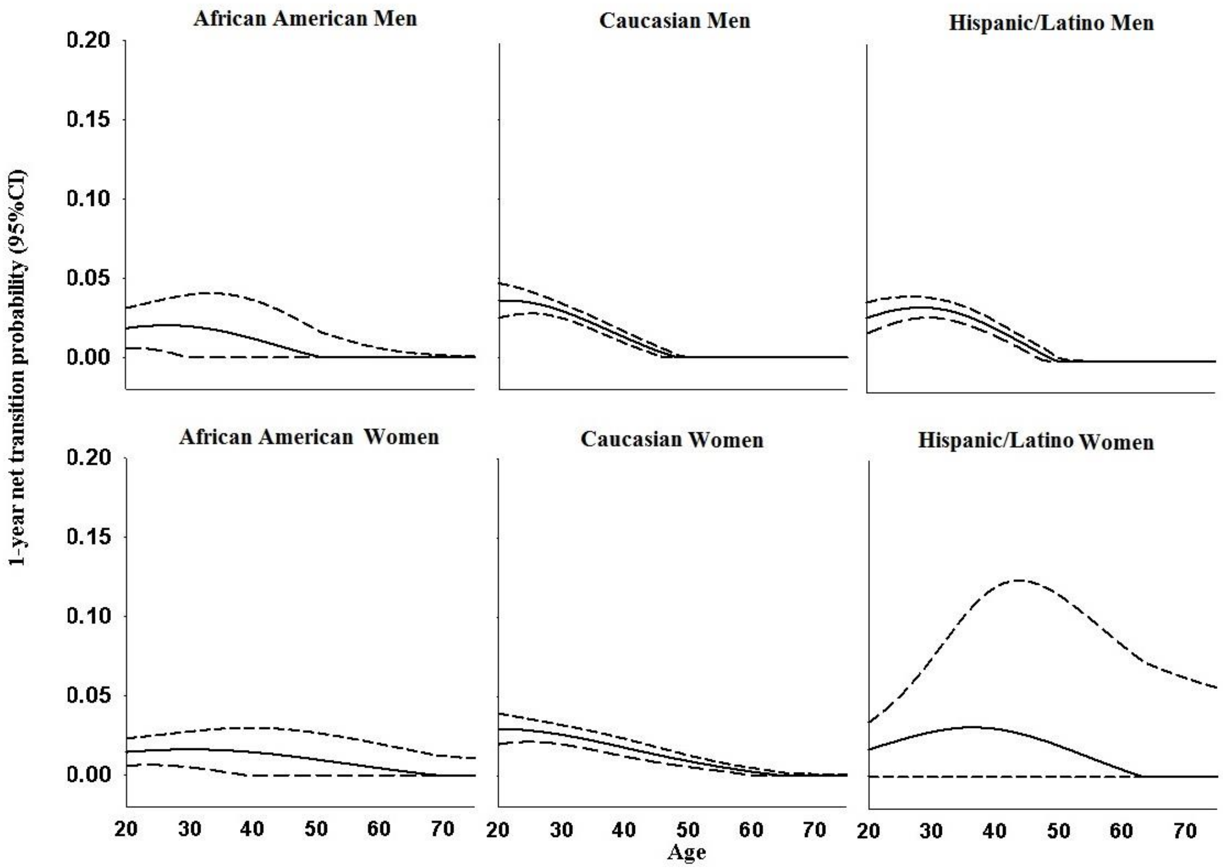

Supplementary Figure 5. Age (20-75)-, race/ethnicity-, and sex-specific ideal LDL-C to intermediate LDL-C net transition probabilities

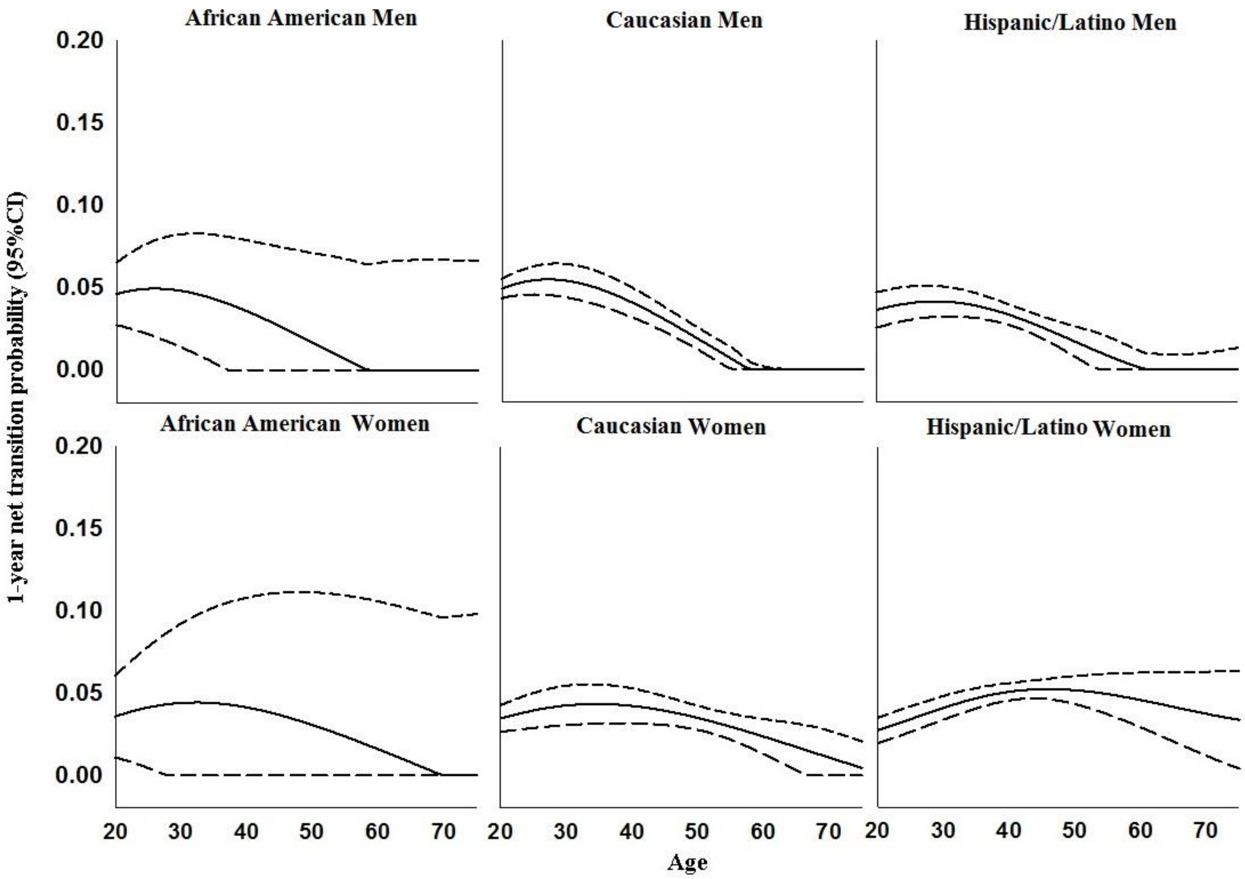

Supplementary Figure 6. Age (20-75)-, race/ethnicity-, and sex-specific intermediate LDL-C to poor LDL-C net transition probabilities

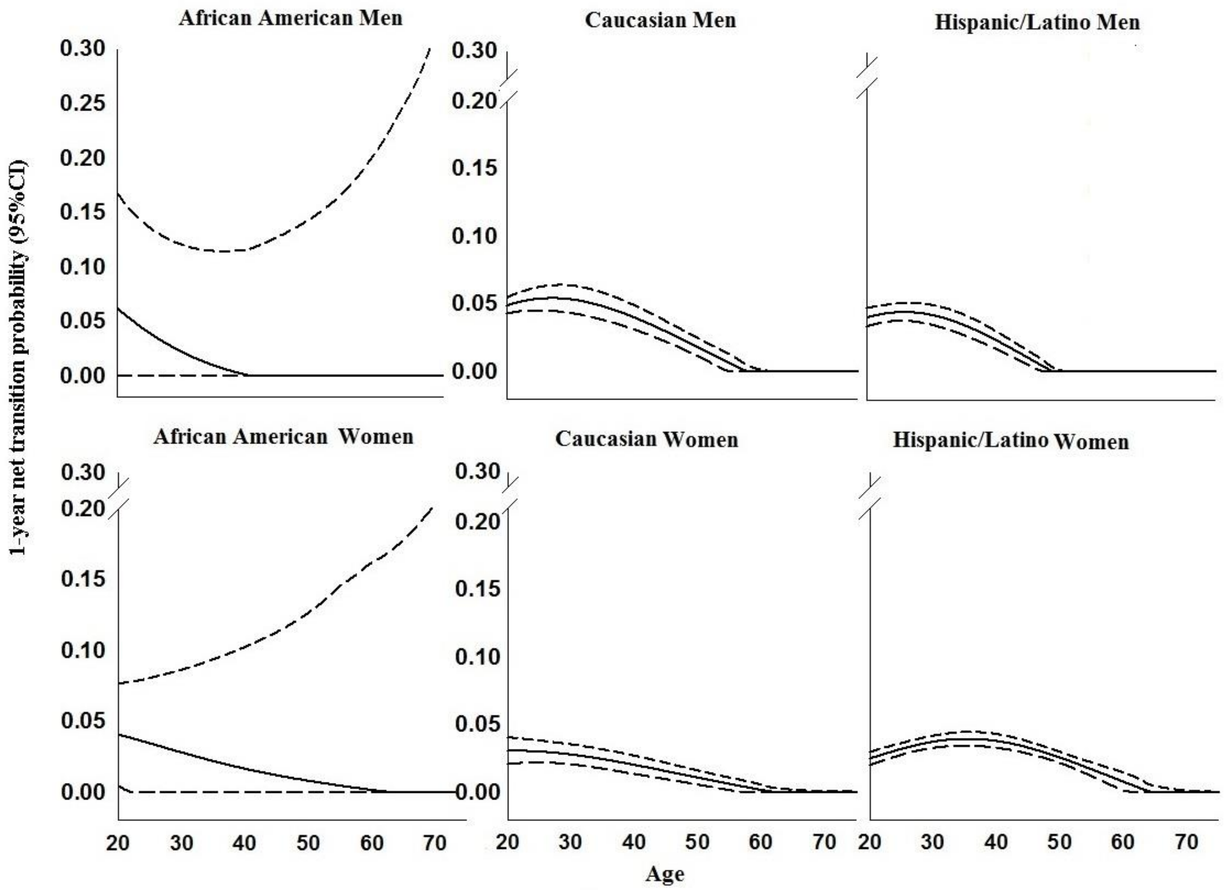

*Page break required to fit African American men net transition probabilities

Supplementary Figure 7. One-year population extrapolations of the net population transitions from ideal-intermediate and intermediate-poor TC and LDL-C

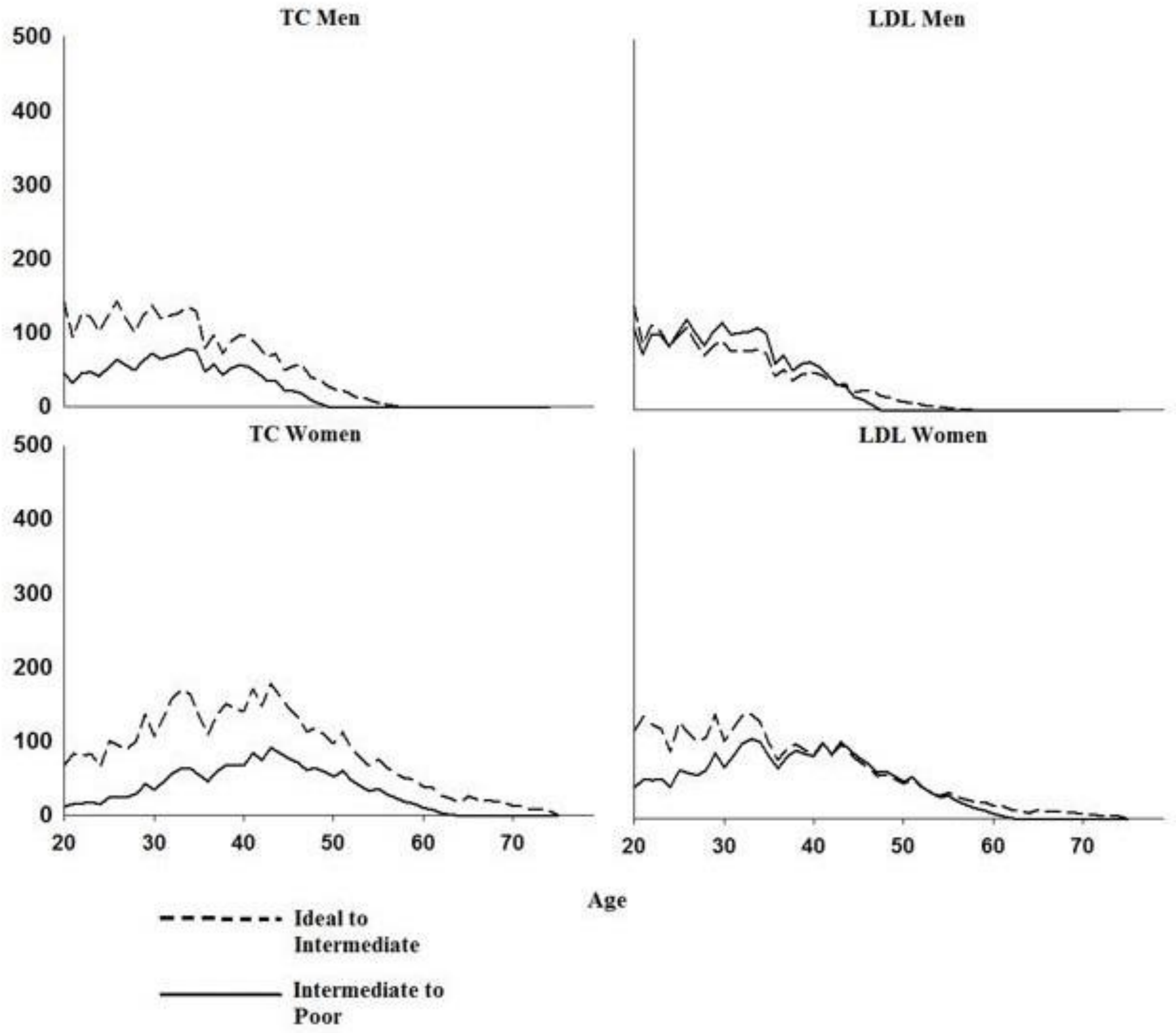

VI. Supplementary references

1. Kassteele J, Hoogenveen RT, Engelfriet PM, Baal PH, Boshuizen HC. Estimating net transition probabilities from cross-sectional data with application to risk factors in chronic disease modeling. *Statistics in medicine*. 2012;31:533-543

2. Friedman GD, Cutter GR, Donahue RP, Hughes GH, Hulley SB, Jacobs DR, Jr., Liu K, Savage PJ. Cardia: Study design, recruitment, and some characteristics of the examined subjects. *Journal of clinical epidemiology*. 1988;41:1105-1116

3. Golub GH, Welsch JH. Calculation of gauss quadrature rules. *Mathematics of Computation*. 1969;23:221-230
